# Supplementary material for: Infrared driven hot electron generation and transfer from non-noble metal plasmonic nanocrystals
Source: Nat Commun. 2020 Jun 10;11:2944. doi: 10.1038/s41467-020-16833-1 (PMC7287091; doi:10.1038/s41467-020-16833-1)
Supplement: Supplementary file 1 — Supplementary Information [file 41467_2020_16833_MOESM1_ESM.docx]

Supplementary Information

**Infrared Driven Hot Electron Generation and Transfer from Non-Noble Metal Plasmonic Nanocrystals**

Zhou et al.


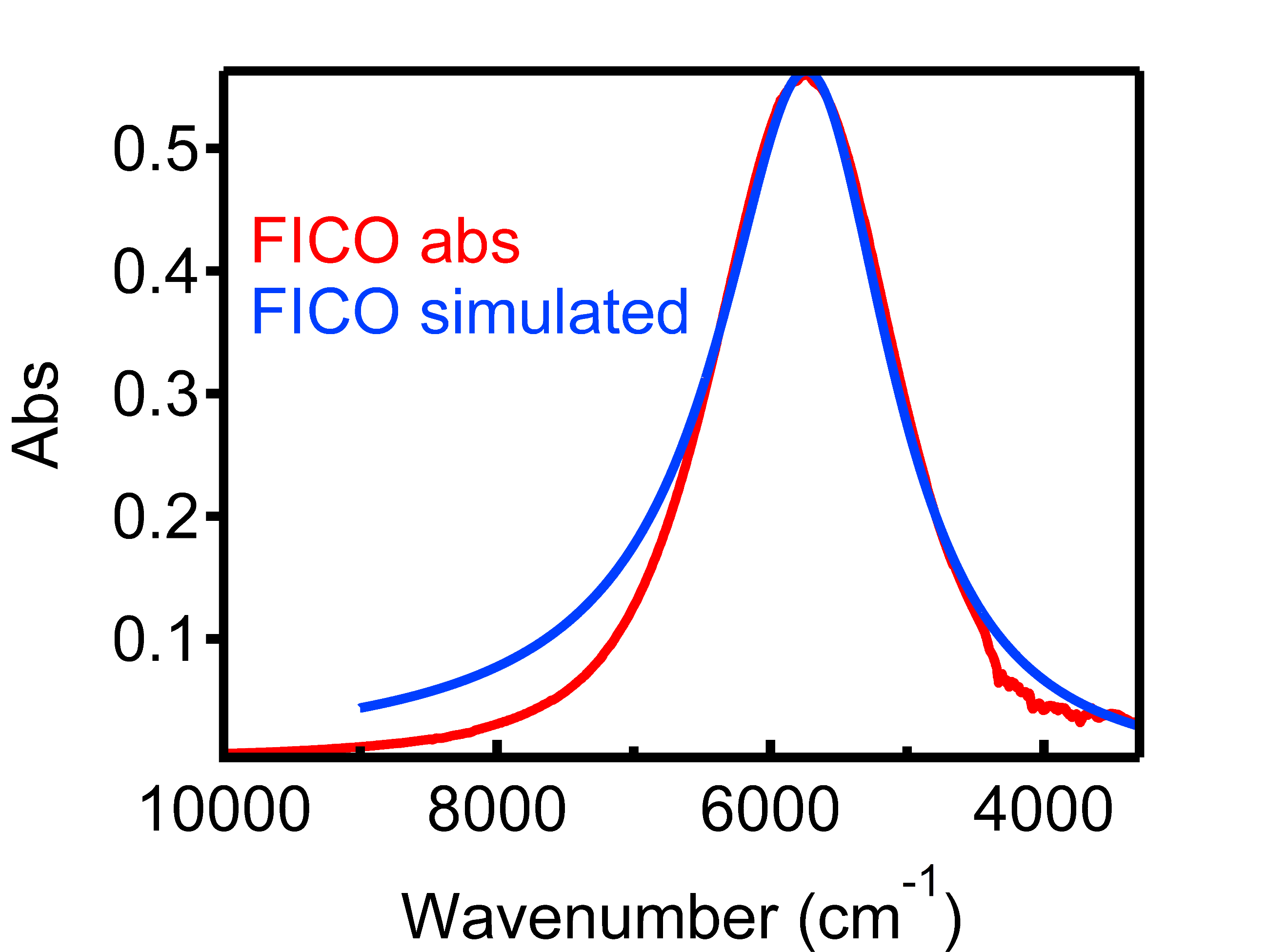


Supplementary Figure 1: Experimental and simulated plasmon absorption of FICO NCs


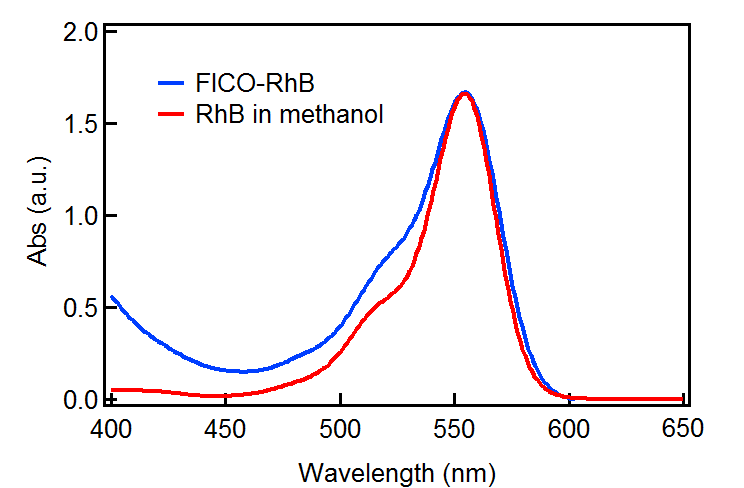


Supplementary Figure 2: Comparison of absorption spectra of FICO-RhB and RhB in methanol solution. The wavelength of later has been redshifted by 7 nm to account for solvent. The peak width is similar.


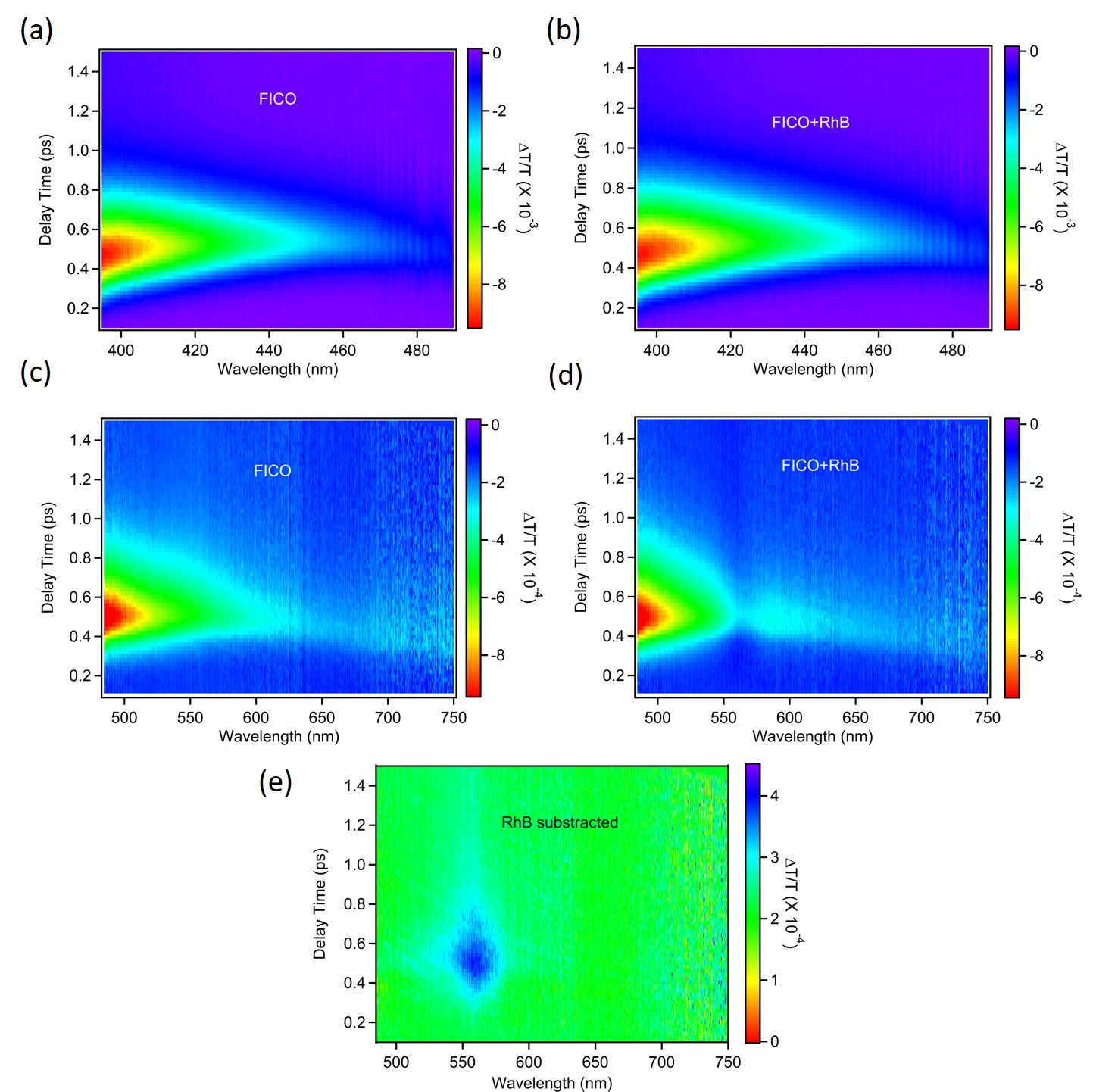


Supplementary Figure 3. 2D fs-TA spectra for FICO NCs in (a) 390-490 nm and (c) 500-750 nm, FICO-RhB in (b) 390-490 nm and (d) 500-750 nm FICO and (e) subtracted RhB signal for pumping at 1650 nm.


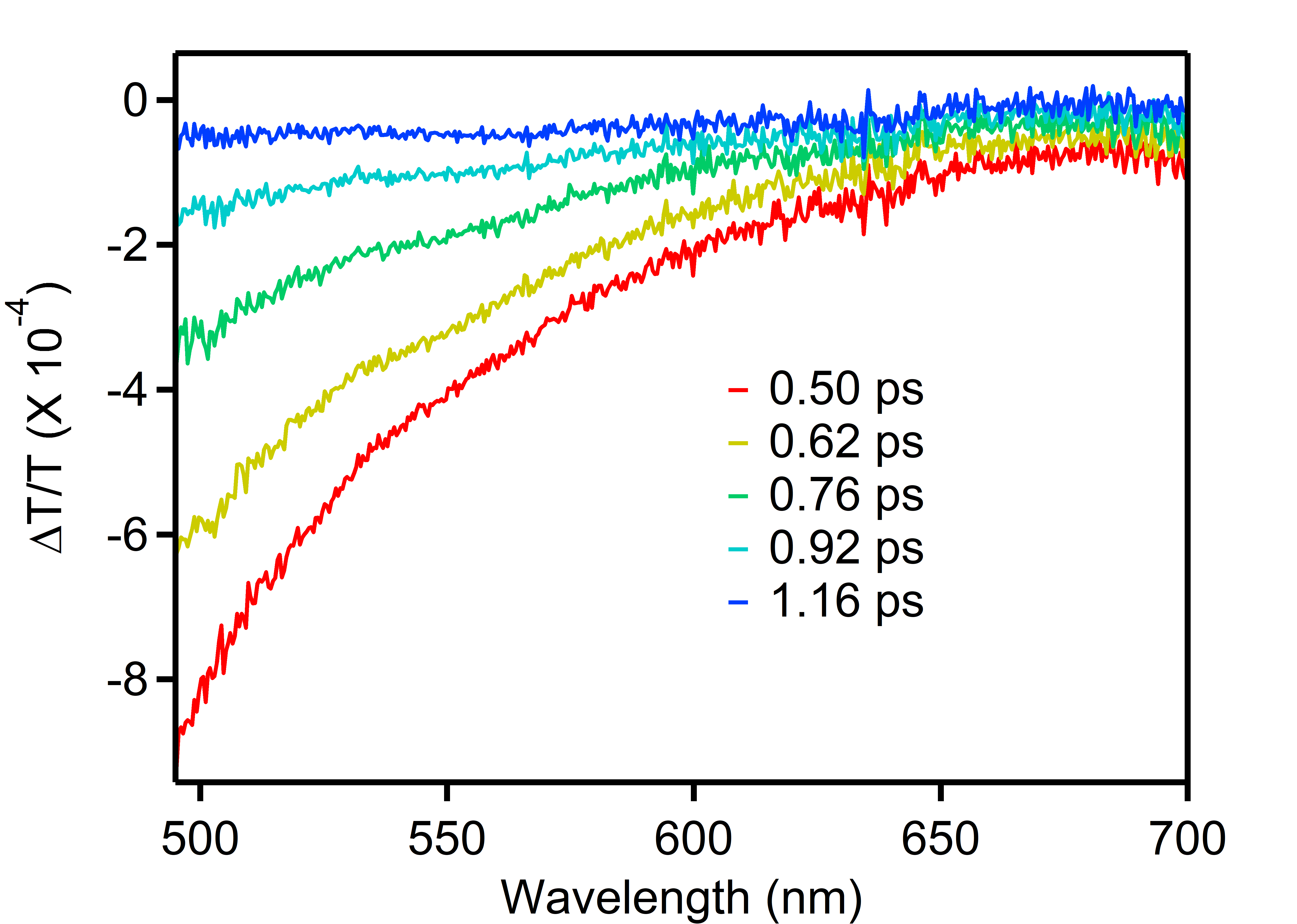


Supplementary Figure 4. Induced absorption at different delay time after e-e scattering for FICO NCs.


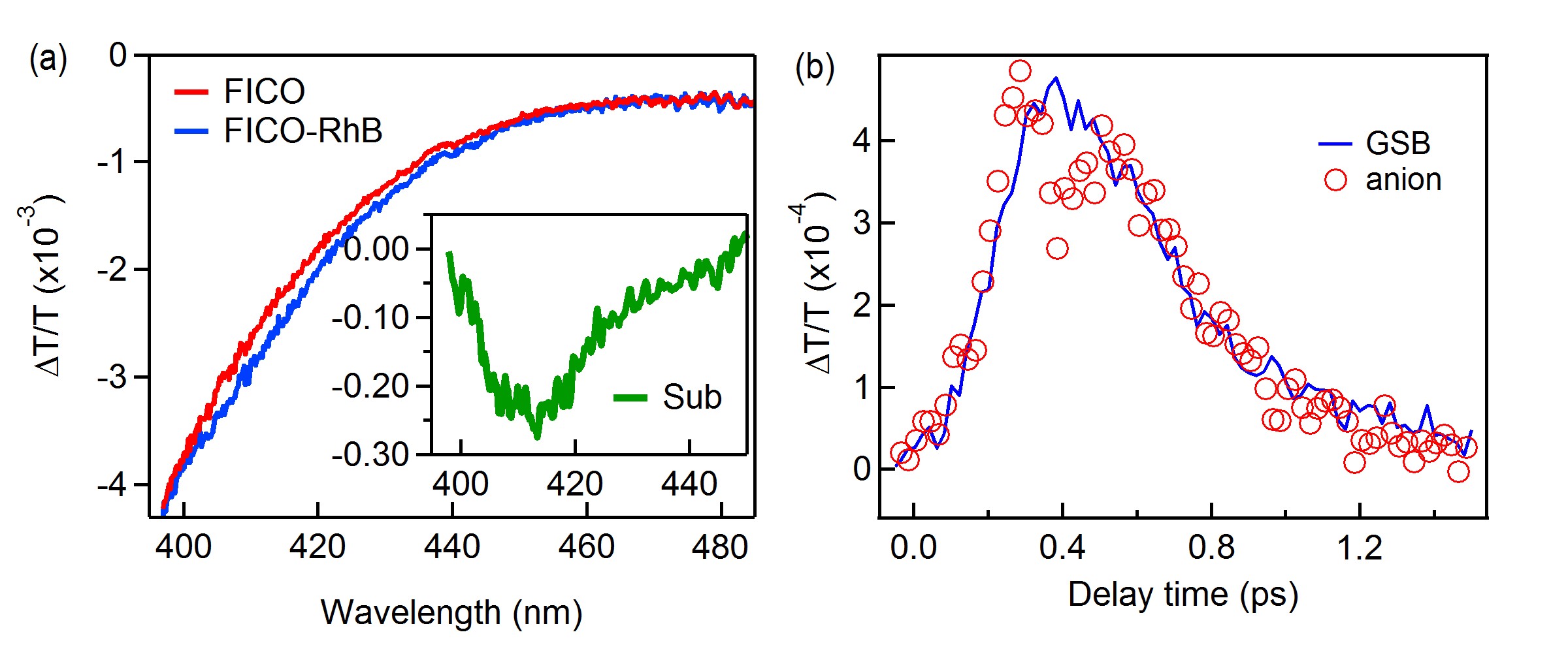


Supplementary Figure 5. (a) Subtracting 0.4 ps TA spectrum of FICO-RhB by that of FICO yields a difference spectrum of RhB anion radical (inset). (b) Comparison between GSB and anion of RhB.


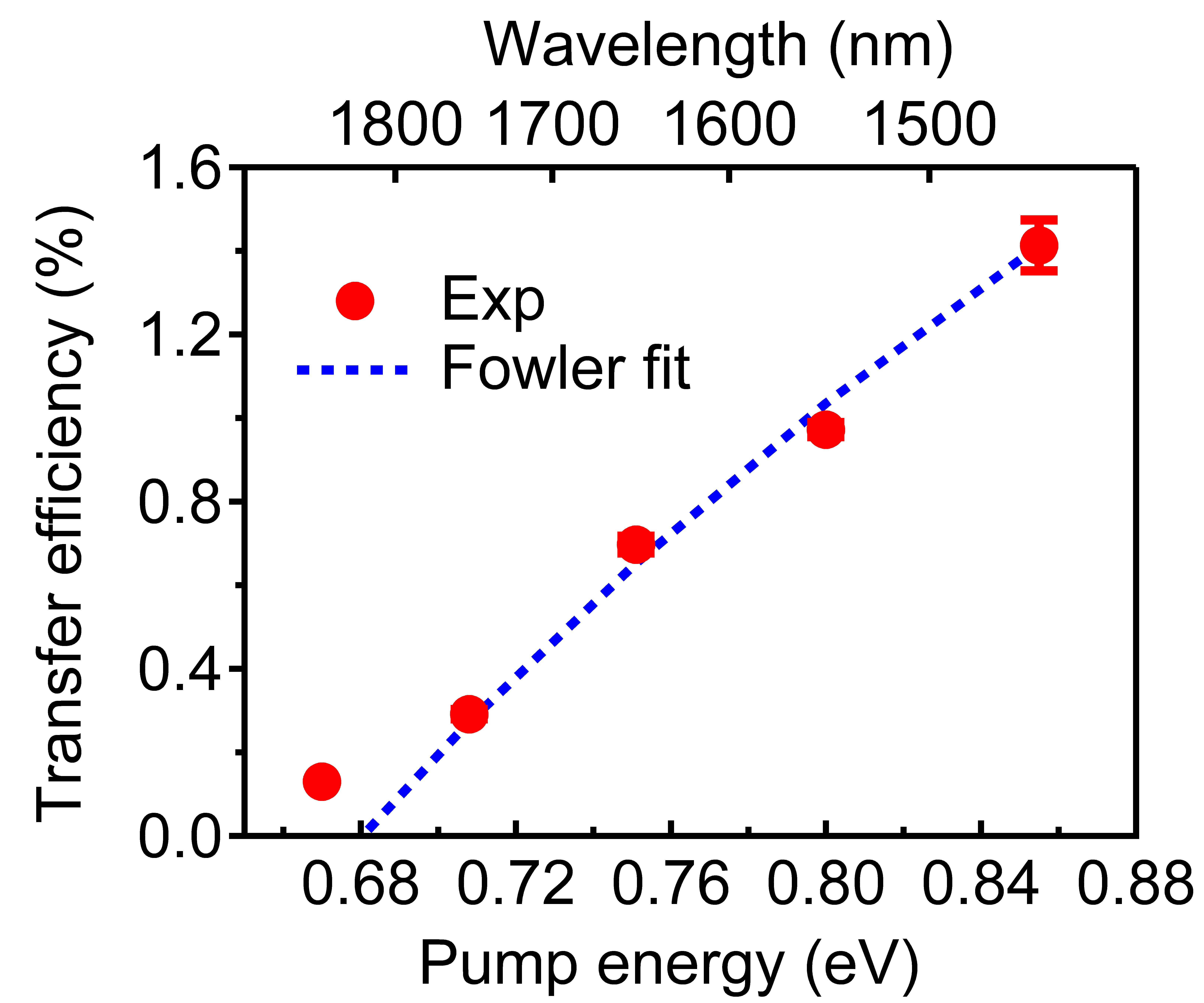


Supplementary Figure 6. Fowler fit of pump energy dependent PIHET QY

Supplementary Figure 7. Pump-probe cross correlation function (IRF) and Gaussian fit with a FWHM ~ 0.2 ps.

**Supplementary Note 1. Plasmon absorption is calculated** by Mathematica using Mie theory.[^1^](#_ENREF_1)

(1) $\sigma_{\mathrm{abs}}=3V\mathbf{k}\sqrt{\varepsilon_{m}}\text{Imag}\left\{ \frac{\varepsilon_{p}-\varepsilon_{\boldsymbol{m}}}{\varepsilon_{p}+2\varepsilon_{m}} \right\}$

The calculation needs the volume of FICO NCs (*V*), the wavevector (**k**), the dielectric constant of the medium ($\varepsilon_{m}$) ie. Hexane, and the dielectric function of NCs ($\varepsilon_{p}$).

The frequency dependent dielectric function of FICO follows the Drude model.

(2) $\varepsilon_{p}(\omega)=\varepsilon_{\infty}-\frac{\omega_{p}^{2}}{\omega^{2}+i\omega\Gamma}$.

$\varepsilon_{\infty}$ refers to the high frequency dielectric constant of CdO, *Γ* is the electronic damping constant and the plasma frequency $\omega_{p}$ depends on the carrier concentration *n_e_*, the carrier effective mass *m*, the electron charge *e* and the permittivity of free space $\varepsilon_{0}$,

(3) $\omega_{p}=\sqrt{\frac{n_{e}e^{2}}{\varepsilon_{0}m}}$

The absolute plasmon absorption spectrum can be fitted using the Beer-Lambert law,

(4) $A_{\mathrm{mie}}=\frac{N\sigma_{\mathrm{abs}}L}{ln(10)}$

Here, *N* refers to the NC density, $N=\frac{f_{V}}{V}$, with *f*_V_ representing the volume fraction, ie, the FICO volume percentage in the absorption measurement.

After inputting the volume of FICO NC (*V*), the dielectric constant of hexane ($\varepsilon_{m}=1.89$), the electron effective mass in CdO (*m* = 0.35*m*_e_), the high frequency dielectric constant of CdO ($\varepsilon_{\infty}=4.8$) and the pathlength of the cuvette (*L* = 1mm), the fitting could help to estimate the carrier concentration *n_e_*, the damping constant *Γ* and the volume fraction *f*_V_. These parameters could further be used to calculate the electron temperature *T*_e_ as discussed below.

|  | ε_∞_ | ω_p_ (cm^-1^) | Γ (cm^-1^) | m/m_e_ | n_e_ (cm^-3^) | *f*_v_ |
| --- | --- | --- | --- | --- | --- | --- |
| FICO | 4.8^a^ | 16811 | 1550 | 0.35^b^ | 1.11×10^21^ | 3.6×10^-5^ |
| FICO-RhB | 4.8 | 16527 | 1700 | 0.35 | 1.07×10^21^ | 3.8×10^-5^ |

Supplementary Table 1: The parameters derived from the plasmon simulation. a and b are the high frequency dielectric constant and effective electron mass at high carrier concentration(10^21^ cm^-3^).[^2^](#_ENREF_2)^,^ [^3^](#_ENREF_3)

**Supplementary Note 2. The number of RhB attached to each FICO NC can be calculated by** dividing RhB concentration (c_RhB_) by FICO concentration (c_FICO_))in solution.

(5) $n^{0}=\frac{c_{\mathrm{RhB}}}{c_{\mathrm{FICO}}}$

c_RhB_ is calculated based on the absorption and absorption coefficient of RhB when mixing with FICO in hexane, and we have c_RhB_ = 7.7*10^-6^ mol L^-1^. c_FICO_ is calculated based on the volume fraction (*f_v_*) from plasmon simulation. As shown in supplementary Note 1 table 1, we have *f*_v_ = 3.8×10^-5^, this correspond to the NC concentration as c_FICO_ = 8.35*10^-8^ mol L^-1^ with NC size d = 11.3 nm inputted. Thus the number of RhB attached to each FICO NC is 92.2.

**Supplementary Note 3. Induced absorption at certain decay time can be modeled** by multiplying the Fermi Dirac distribution at certain electron temperature *T*_e_ with the relative DOS $\rho\left( \varepsilon\right)$ for each energy (wavelength).

The Fermi-Dirac distribution is calculated by

(6) $f\left( \varepsilon\right)=\frac{1}{1+e^{(\varepsilon-E_{f})/kT_{e}}}$

*E_f_* is at -4.62 eV, *T*_e_ is the input electron temperature.

Band dispersion for conduction band is using parabolic assumption, thus the relative DOS $\rho\left( \varepsilon\right)$ can be calculated by

(7) $\rho\left( \varepsilon\right)=\sqrt{\varepsilon+6.25}$

The energy level of *E_CBM_* is at -6.25 eV.

The induced absorption signal can be fitted by

(8) $\frac{\Delta T}{T}\left( \varepsilon\right)=-\left( 1-f\left( \varepsilon\right) \right)*\rho\left( \varepsilon\right)$

As noted in the main text, valance band can be regarded as flat, so the induced absorption can be treated only by the unoccupancy of the conduction band. Based on the fitting, we can extract the *T_e_* for different decay time after electron thermalization.

**Supplementary Note 4. Theoretical maximum electron temperature *T_e,max_* after Fermi Dirac distribution considering no energy loss can be calculated by** total energy absorbed by FICO NCs in cuvette divided by the electron heat capacity $\gamma T_{e}$ at room temperature and NC volume:[^1^](#_ENREF_1)

(9) $T_{e,max}=\frac{FA(\omega)}{L\gamma T_{e}f_{V}}$

Where *F* is the pump fluence, *A(ω)* is the absorption fraction at *ω*, *L* is the optical path length.

For electron heat capacity $\gamma T_{e}$, *γ* is calculated based on the local density of states (DOS) at the Fermi level and can be further determined by:

(10) $\frac{\gamma_{1}}{\gamma_{2}}=\frac{m_{1}{n_{e,1}}^{1/3}}{m_{2}{n_{e,2}}^{1/3}}$

$\gamma T_{e}$value for metals is known, such as Ag, once the effective mass and carrier concentration *n*_e_ is determined from the plasmon absorption simulation, $\gamma T_{e}$ for FICO NCs can be calculated, so as the maximum electron temperature *T*_e,max_.

**Supplementary Note 5. Experimental PIHET quantum yield (QY) can be calculated by** the number of bleached RhB molecular divided by the total photon absorbed by the FICO NCs in the overlap volume (*V*) of pump and probe laser.

(11) $\phi=\frac{n_{RhB}}{n_{photon}}$

$n_{RhB}$ was calculated by:

(12) $n_{RhB}=\frac{lg({(\frac{dT}{T})}_{@554nm}+1)}{{L_{overlap}^{probe}\varepsilon}_{@554nm}}V_{overlap}N_{A}$

$lg({(\frac{dT}{T})}_{@554nm}+1)$ is the change of absorption of RhB, ie *A – A^’^*. *L*_overlap_^probe^ is the probe optical path length in the overlap region, $\varepsilon_{@554nm}=1.15\times{10}^{5}{mol}^{-1}L{cm}^{-1}$ for RhB.[^4^](#_ENREF_4) *N*_A_ is the Avogadro constant.

$n_{\mathrm{photon}}$ was calculated by:

(13) $n_{\mathrm{photon}}=\frac{AE_{\mathrm{pulse}}}{E\left( \omega\right)}(1-{10}^{-Abs@\omega})\frac{L_{\mathrm{overlap}}^{p\mathrm{ump}}}{L}$

*A* is the fraction of the beam intensity within the FWHM of laser pulse, i.e. 0.5. $E_{\mathrm{pulse}}$ is the energy per pulse, $E\left( \omega\right)$ is the energy of single photon at pump wavelength, $Abs@\omega$ is the absorption of FICO at pump wavelength in cuvette, *L*_overlap_^pump^ is the pump optical path length in the overlap region.

**Supplementary Note 6. Percentage of hot electron higher than the LUMO of RhB *after* electron thermalization for different pump fluences is estimated** based on the Fermi-Dirac distribution $f\left( \varepsilon\right)$for each *T_e_* at certain pump fluence and the relative DOS $\rho(\varepsilon)$ near the Fermi level, the percentage $\phi$ can be calculated by the number of electrons higher than LUMO level of RhB divided by the total electrons:

(14) $\phi=\frac{\int_{-3.94}^{\infty} \rho(\varepsilon)f\left( \varepsilon\right)d\varepsilon}{\int_{-6.25}^{\infty} \rho(\varepsilon)f\left( \varepsilon\right)d\varepsilon}$

Where -3.94 eV is the LUMO of RhB and -6.25 eV is the *E*_CBM_.

**Supplementary Note 7. Averaged number of photons absorbed per FICO NC can be calculated as follows:**

(15) $n_{photon per NC}=\frac{\frac{FS}{\xi\left( \omega\right)}(1-{10}^{-Abs@\omega})}{\frac{f_{V}V_{\mathrm{total}}}{V_{\mathrm{NC}}}}$

*F* is the pump fluence, S is the area unit. $\xi\left( \omega\right)$ is the energy (μJ) of photon at $\omega$. *f*_V_ is the volume fraction discussed in supplementary note 1. $V_{\mathrm{total}}$ is the total pump volume in in 1mm cuvette in unit area. $V_{\mathrm{NC}}$ is the FICO nanocrystal volume. The numerator essentially calculates the number of photons at $\omega$ been absorbed by FICO NCs in 1mm cuvette in unit area. The denominator calculates the number of FICO NCs in 1mm cuvette in unit area.

**Supplementary Note 8. PIHET QY can be modeled through** calculating the percentage (γ) of electrons above RhB LUMO right after Landau damping and multiply by the transfer efficiency$\eta$. The hot electron distribution right after Landau damping is depicted based on the energy conservation and parabolic band dispersion near the Fermi level, thus γ can be calculated as:

(16) $\gamma=\frac{\int_{E_{f}-(\hbar\omega-E_{b})}^{\infty} \rho\left( \varepsilon\right)f\left( \varepsilon\right)*\rho(\varepsilon+\hbar\omega)(1-f\left( \varepsilon+\hbar\omega\right))d\varepsilon}{\int_{E_{f}-1.63}^{\infty} \rho\left( \varepsilon\right)f\left( \varepsilon\right)*\rho(\varepsilon+\hbar\omega)(1-f\left( \varepsilon+\hbar\omega\right))d\varepsilon}$

*E_b_* is the barrier between the LUMO of RhB and Fermi level (*E*_f_), $\rho(\varepsilon)$ is the density of state at $\varepsilon$ with parabolic band dispersion assumption, $f\left( \varepsilon\right)$ is the Fermi-Dirac distribution at $\varepsilon$ with *T*_e_ = 300K. The numerator is calculating the number of hot electrons with energy higher than the LUMO, the denominator is calculating the total hot electron generated.

(17) $QY=\eta\gamma$

We can fit the experimental value of pump energy dependent transfer efficiency with our model to get the best fit *E_b_*. With 1450 nm pump, the γ is estimated to be 25.9%, the portion of electrons above barrier that can be transferred ($\eta$) was estimated to be 5.5%.

We also noted that the transfer quantum efficiency across the Schottky junction from metal into semiconductor is often depicted using Fowler equation.[^5^](#_ENREF_5)

(18) $QY\propto\frac{{(\hbar\omega-eE_{b})}^{n}}{eE_{f}\hbar\omega}$

n is a material determined parameter. We can also fit our data with Fowler equation and get n = 1 (see Fig S4). Fowler fit with n = 1 is used in the system where electron linear momentum is relaxed due the the roughness of the junction.[^6^](#_ENREF_6) We would expect the non-conservation of momentum for hot electron transfer in our system with molecues attached on the nanocrystal surface. The other difference between our model and Fowler equation are as follows:

1. In derivation of Fowler equation, hot electrons with different injection angles to the junction surface are integrated. Since the interface of our system is not a solid junctoin surface, we do not need to consider the injection angle.
2. The density of states $\rho(\varepsilon)$ in Fowler equation’s derivation is using the DOS at Fermi surface, i.e. $\rho\left( \varepsilon\right)\approx\rho\left( E_{F} \right)$. This assumpiont is solid when the Fermi surface is much higher than the conduction band minimun ($E_{\mathrm{CBM}})$. In our system, the Fermi level is only 1.63 eV higher than $E_{CBM}$, thus we are using the parabolic assuption for the conduction band dispersion to calculate the DOS.
3. Our model also includes the room temperature Fermi-Dirac distribution for states near Fermi level. And we integrate from the bottom of conduction band to infinity thus all the possible occupied states and empty states can be counted. This allows us to calculated the effeciecy of pump photons with energy lower than E_b_.

**Supplementary reference**

1. Johns RW*, et al.* Charge carrier concentration dependence of ultrafast plasmonic relaxation in conducting metal oxide nanocrystals. *J. Mater. Chem. C* **5**, 5757-5763 (2017).

2. Liu CP*, et al.* Effects of Free Carriers on the Optical Properties of Doped CdO for Full-Spectrum Photovoltaics. *Phys. Rev. Appl.* **6**, 064018 (2016).

3. Zhu Y, Mendelsberg RJ, Zhu J, Han J, Anders A. Dopant-induced band filling and bandgap renormalization in CdO : In films. *J. Phys. D* **46**, 195102 (2013).

4. Farag AAM, Yahia IS. Structural, absorption and optical dispersion characteristics of rhodamine B thin films prepared by drop casting technique. *Opt. Commun.* **283**, 4310-4317 (2010).

5. Fowler RH. The Analysis of Photoelectric Sensitivity Curves for Clean Metals at Various Temperatures. *Physical Review* **38**, 45-56 (1931).

6. Giugni A*, et al.* Hot-electron nanoscopy using adiabatic compression of surface plasmons. *Nat. Nanotechnol.* **8**, 845-852 (2013).
